# Increased Risk of Breast Fibroadenomas Among Obese and Postmenopausal Women With Uterine Fibroids

**DOI:** 10.7759/cureus.43503

**Published:** 2023-08-15

**Authors:** Angham T Nawar, Alya Binmahfouz, Ebrahym Abbas, Smaher F Almehmadi, Sameerah A Turson, Ibrahem H Kanbayti

**Affiliations:** 1 Radiological Sciences, Faculty of Applied Medical Sciences, King Abdulaziz University Hospital, Jeddah, SAU; 2 In Vitro Fertilization Unit, Center for Innovation in Personalized Medicine, King Abdulaziz University Hospital, Jeddah, SAU; 3 Radiology, King Abdulaziz University Hospital, Jeddah, SAU

**Keywords:** age, postmenopausal women, body mass index, breast fibroadenoma, uterine fibroid

## Abstract

Introduction

Uterine fibroids and breast fibroadenomas are common female benign neoplasms that are similarly derived from a single clonal origin and are modulated by estradiol concentration in blood. However, the association between these neoplasms has not yet been explored. Hence, this study aims to investigate the relationship between uterine fibroids and breast fibroadenomas.

Methods

A total of 199 women (cases: 72 women with uterine fibroids, control: 127 women without uterine fibroids) were included in this study. Ultrasound was used to screen for uterine fibroids, and both ultrasound and biopsy were utilized to diagnose breast fibroadenomas. Logistic regression analysis was used to investigate the association between uterine fibroids and breast fibroadenomas and the factors associated with the relationship.

Results

Women with uterine fibroids have more than two times higher odds of having breast fibroadenomas among older women (p=0.03), more than twofold increased odds of breast fibroadenomas among obese females (p=0.05), and higher odds of having breast fibroadenomas among postmenopausal transition participant groups (OR=9.6; 95% CI 1.98-30.14; p-value=0.005).

Conclusion

The association between uterine fibroids and breast fibroadenomas is significantly pronounced among older, obese, and postmenopausal women. This relationship might be driven by the indirect stimulation of estrogen hormone receptors via adipose tissue or other lifestyle as well as genetic factors. Therefore, further larger prospective studies considering these factors are needed to replicate the current findings.

## Introduction

Uterine fibroids and breast fibroadenomas are the most prevalent benign gynecologic disorders in women. The etiology of these disorders is unknown. A potential causative relationship between these benign tumors and the expression of sex hormones in the body has been previously reported [1,2]. The increased prevalence of breast fibroadenomas among younger women is believed to be due to their higher estrogenic activity [3]. Additionally, the administration of hormonal therapy has been found to escalate the risk of developing uterine fibroids among postmenopausal women [4]. Given that breast fibroadenomas and uterine fibroids are driven by female reproductive sex hormones [1, 5], the association between them is reasonable. However, the literature on the relationship between breast fibroadenomas and uterine fibroids is very limited and conflicting, with one study reporting the presence of an association [6] and another that does not [7]. Therefore, further studies are needed to address these discordant findings among the studies.

Evidence exists for the association of these gynecologic benign tumors with demographic, anthropometric, genetic, and hormonal factors [8-10]. Despite this evidence, it is unclear if these factors confound the association between breast fibroadenomas and uterine fibroids. Among the two published studies that sought the relationship between breast fibroadenomas and uterine fibroids, one study did not adjust for body mass index (BMI), menopause status, and age [7]. The study reported no association, which might explain the conflicting findings of the current studies. Thus, future studies are warranted to explore their contributing effect.

Given that the incidence of breast fibroadenomas and uterine fibroids differ across countries due to differences in race, lifestyle, and environmental factors [11,12], the potential link between breast fibroadenomas and uterine fibroids may be distinct for the Saudi population. However, current studies are mainly based on the Western population; therefore, this study aims to explore the association between uterine fibroids and breast fibroadenomas among Saudi women.

## Materials and methods

Study population

A case-control study was conducted with a sample size of 199 subjects. Seventy-two women with at least one uterine fibroid and who attended King Abdul-Aziz University Hospital for pelvic ultrasound checkups were considered cases. The control group included 127 women who attended the hospital for different gynecological causes other than fibroids. Women with diagnosed breast cancer, mastectomy, or hysterectomy were excluded from the current study. The Institutional Review Board (IRB) of the Faculty of Medicine, KAU (Reference No: 37-22), granted approval for the study and waived the requirement for patient consent.

Data collection

Variables such as age, menopause status, BMI, marital status, presence of breast fibroadenomas, and presence of uterine fibroids were retrospectively collected from the hospital information system (HIS). Patients with uterine fibroids were diagnosed by transabdominal pelvic ultrasound with a convex probe low-frequency transducer or by a transvaginal probe. Lumps with ultrasound characteristics of fibroadenomas, between 1 cm and 2.5 cm in size, discovered in younger females with no familial background of breast cancer or histologically confirmed by biopsy among individuals with a highly suspicious profile of malignancy were considered positive for fibroadenomas. The scan results were reported by a consultant breast radiologist.

Statistical analysis

For statistical purposes, frequencies and means were used as appropriate to describe population characteristics. The chi-square test was used to explore the differences between cases and control groups in terms of demographic, anthropometric, and reproductive characteristics. A stratified logistic regression analysis was conducted to examine the effect of age, BMI, and menopause status on the potential relationship between uterine fibroids and breast fibroadenomas. Due to a limited number of younger women (age ≤30 years old), a threshold for age stratification of 45 years was used in the logistic regression analysis. All p-values were two-sided and considered statistically significant if they were ≤ 0.05. All statistics were performed using Statistical Product and Service Solutions (SPSS) for Windows, version 29 (IBM, Armonk, NY, USA).

## Results

A total of 199 females with 72 uterine fibroids were included in this study. The mean age was 44.25 years (SD: ±11.5; range: 19-75 years), and the mean BMI was 28.43 kg/m2 (SD: ±6.36; range: 16-49 kg/m2). Most women were married (71.9%) and premenopausal (66.3%). Nearly half of all the participants were older (47.2%), and 33.8% were obese. Only 36.4% of females were diagnosed with breast fibroadenoma (Table 1).

**Table 1:** Population characteristics SD: standard deviation; BMI: body mass index; n: number of participants

Population characteristics		n (%)
Age	Less than or equal to 30 years	28 (14.4)
	Between 31 and 45 years	77 (38.7)
	More than or equal to 46 years	94 (47.2)
Presence of uterine fibroid	Women with uterine fibroids	72 (36.2)
	Women without uterine fibroids	127 (63.8)
Presence of breast fibroadenoma	Women with breast fibroadenoma	72 (36.1)
	Women without breast fibroadenoma	126 (63.3)
	Missing	1 (0.58)
Body mass index (BMI)	Underweight	6 (3)
	Normal weight	59 (29.6)
	Overweight	64 (32.1)
	Obese	67 (33.6)
	Missing	3 (1.6)
Menopause status	Premenopausal	132 (66.3)
	Postmenopausal	67 (33.7)
Married status	Married	143 (71.9)
	Not married	33 (16.6)
	Missing	23 (11.6)
Age mean (±SD) years	44.25 (SD: ±11.5; range: 19-75 years)	
BMI mean (±SD) kg/m^2^	28.43 (SD: ±6.36; range: 16-49 kg/m^2^)	

Patients with uterine fibroid were more likely to be older (p=0.01) and among the postmenopausal women group (p<0.001). None of the other population characteristics including BMI, and the presence of fibroadenoma showed significant differences between cases and control groups (p≥0.06) (Table 2).

**Table 2:** Characteristics of women in cases and control group BMI: body mass index; n: number of participants

Patient characteristics		Cases n (%)	Control n (%)	p-value
Presence of fibroadenoma	Women with breast fibroadenoma	31 (43.6)	41 (32.2)	0.1
	Women without breast fibroadenoma	40 (56.3)	86 (67.7)	
Age	Less or equal to 30 years old	3 (4.16)	25 (19.6)	0.01
	Between 31-45	23 (31.9)	54 (42.5)	
	More than or equal to 46 years old	46 (63.8)	48 (37.7)	
Menopausal status	Premenopausal	36 (50)	96 (75.6)	<0.001
	Postmenopausal	36 (50)	31 (24.4)	
BMI	Normal range	18 (25)	41 (32.5)	0.23
	Overweight	25 (34.7)	39 (31)	
	Obese	28 (38.9)	39 (31)	
Marital status	Married	53 (73.6)	90 (70.9)	0.4
	Single	9 (12.5)	24 (18.9)	

In univariate logistic regression analysis, there was no significant heterogeneity in the relationship between uterine fibroids and fibroadenomas (p=0.11) (Table 3).

**Table 3:** Odds ratios of fibroadenomas in relation to uterine fibroids OR: odds ratio; 95% CI: 95% confidence interval

Presence of uterine fibroids	Presence of breast fibroadenomas OR (95% CI)	p-value
Women without uterine fibroids	1 (reference)	0.11
Women with uterine fibroids	1.62 (0.89-2.95)	

According to the univariate logistic regression analysis stratified by population characteristics, there were more than two times higher odds of having breast fibroadenomas among older women with uterine fibroids compared to those without (p=0.03). Obese women with uterine fibroids had more than a twofold increased odds of breast fibroadenomas (p=0.05). Relative to premenopausal women with uterine fibroids, those who were among the postmenopausal women group had higher odds of having breast fibroadenomas (OR=9.6; 95% CI=1.98-30.14; p-value=0.005) (Table 4).

**Table 4:** Odds ratios of fibroadenomas in relation to uterine fibroid by population characteristics including age, BMI, and menopause status BMI: body mass index; OR: odds ratio; 95% CI: 95% confidence interval

Population characteristics			Presence of breast fibroadenoma OR (95% CI)		Absence of breast fibroadenoma OR (95% CI)	p-value
Women with uterine fibroids	Age	Less than and equal to 45 years	2.11 (0.85-5.24)		0.47 (0.19-1.17)	0.1
		More than or equal to 46 years old	2.92 (1.06-2.08)	0.34 (0.12-0.94)		0.03
	BMI	Normal weight	1.37 (0.43-4.34)		0.73 (0.23-2.31)	0.5
		Overweight	1.76 (0.62-5)		0.56 (0.20-1.60)	0.28
		Obese	2.69 (1.95-7.63)		0.37 (0.13-0.95)	0.05
	Menopause status	Premenopausal	1.30 (0.60-2.82)		0.76 (0.35-1.65)	0.4
		postmenopausal	9.6 ( 1.98-30.14)		0.1 (0.02-0.5)	0.005

## Discussion

Our findings show that uterine fibroids are significantly associated with breast fibroadenoma among certain categories of women. These findings suggest that several complex potential pathways are in favor of the effect of hormonal factors on the relationship between breast fibroadenomas and uterine fibroids. Importantly, the biosynthesis of hormones is different among women. Younger premenopausal women mainly have estrogen from their ovaries, while obese postmenopausal females synthesize estrogen from adipose tissue using aromatase enzymes existing in fat [13,14].

The potential link between uterine fibroids and breast fibroadenomas has been investigated by previous case-control studies, but with conflicting findings. Although Moini et al.'s study, which included 610 Iranian women and explored the relationship between uterine fibroids and breast fibroadenomas, found no evidence of association [7], Spinos et al.'s study, conducted among the Greek population (120 women), concluded that women with uterine fibroids are frequently diagnosed with breast fibroadenoma [6]. Our work differs from the prior reported studies in that the odds ratios of breast fibroadenomas among women with uterine fibroids were stratified by demographic and anthropometric factors to explore the relationship between these two neoplasms. Doing so has led us to explore which categories of women experience the coexistence of uterine fibroids and breast fibroadenomas.

It was evident that the key agent of an increased risk of breast fibroadenomas and uterine fibroids is related to an increased amount of body fat, which is known as the main source of sex hormone receptor biosynthesis among obese postmenopausal women [5,14]. Higher estrogenic activity induced by the aromatase enzyme in adipose tissue of these categories of women may promote the growth of these benign neoplasms. Therefore, it is plausible that the association between uterine fibroids and breast fibroadenomas is significantly pronounced among postmenopausal and obese females. Interestingly, the findings of the current study support this hypothesis where the odds of having breast fibroadenoma among women with uterine fibroids were found to be significantly higher among obese and postmenopausal women.

It is well established that breast fibroadenoma is common among women in their 20s, whereas uterine fibroids are prevalent in women in their 40s [15,16]. Additionally, these benign diseases are known to decrease in size with age and menopausal status, suggesting that they are modulated by endogenous sex hormone concentration in the blood that gradually drops during perimenopause and menopause stages [2,17]. In the present study, however, it is found that both uterine fibroids and breast fibroadenomas coexisted among older women, categories of women known to have lower estrogenic activity in their bodies. One potential explanation for these discordant findings is that these neoplasms have a multifactorial nature where several factors such as hormonal replacement therapy administration, coffee consumption, smoking, race, and genetic factors may escalate estrogen circulating levels in the bloodstream, which, in turn, contribute to the development of these lesions among these categories of women. Unfortunately, data on these factors were not available, and, therefore, further studies considering these factors are needed to confirm the relationship between uterine fibroids and breast fibroadenomas among these categories of women. Additionally, it is important to note that the date of diagnosis that was considered in the current study does not necessarily reflect the actual date of lesion development. Therefore, the other explanation for the coexistence of both lesions among elderly women could be attributed to benign lesions that developed earlier among asymptomatic women and kept growing during old age due to the factors mentioned above.

Currently, there is no clear scientific evidence to suggest that marital status has a role in the pathophysiology of fibroids and fibroadenomas. However, it is important to note that married women have been shown to have significantly higher levels of estradiol and progesterone hormones than unmarried women [18]. Additionally, it is well-established that the growth and proliferation of breast fibroadenomas and uterine fibroids are stimulated by hormones including estrogen and progesterone [1,2]. Not surprisingly, therefore, the factor of marital status has contributed to the pathogenesis of uterine fibroids and breast fibroadenomas. However, the current study is unable to confirm this hypothesis due to the small number of single women included in the study. Therefore, larger future studies are needed to explore the role of this factor on the etiology of uterine fibroids and breast fibroadenomas.

Fibroadenomas and uterine fibroids can poorly affect a patient’s quality of life if they are lately diagnosed, and fibroadenomas have been found to confer a slight risk of developing malignant tumors in the future [19-21]. Therefore, if our findings are confirmed by larger prospective studies, this would help to improve the knowledge of the underlying pathophysiology of the association between uterine fibroids and breast fibroadenomas. Additionally, it should help inform decision-making regarding the management and screening of patients with these benign tumors.

While the findings of this study are encouraging, it is important that they be interpreted with caution due to several limitations including small sample size, reliant on an inaccurate date of neoplasm diagnosis, the lack of histopathology in all cases of uterine fibroids and some cases of fibroadenomas, and incomplete data on other covariates including hormonal replacement therapy administration, coffee consumption, smoking, race, status of endogenous sex hormone status, and genetic factors.

## Conclusions

The association between uterine fibroids and breast fibroadenomas is significantly pronounced among obese women. Although breast fibroadenomas and uterine fibroids are associated differently with age, this study found that both lesions were present among older and postmenopausal women. Despite the lack of clarity about the mechanisms behind this relationship, genetic and hormonal elements may still play a part. Therefore, further studies considering these factors are needed to confirm our findings.

## References

[1] Ajmal M, Khan M, Van Fossen K (2022). Breast fibroadenoma. StatPearls.

[2] Pavone D, Clemenza S, Sorbi F, Fambrini M, Petraglia F (2018). Epidemiology and risk factors of uterine fibroids. Best Pract Res Clin Obstet Gynaecol.

[3] Houssami N, Cheung MN, Dixon JM (2001). Fibroadenoma of the breast. Med J Aust.

[4] Furness S, Roberts H, Marjoribanks J, Lethaby A (2012). Hormone therapy in postmenopausal women and risk of endometrial hyperplasia. Cochrane Database Syst Rev.

[5] Yang Q, Ciebiera M, Bariani MV, Ali M, Elkafas H, Boyer TG, Al-Hendy A (2022). Comprehensive review of uterine fibroids: developmental origin, pathogenesis, and treatment. Endocr Rev.

[6] Spinos N, Terzis G, Crysanthopoulou A (2007). Increased frequency of thyroid nodules and breast fibroadenomas in women with uterine fibroids. Thyroid.

[7] Moini A, Eslami B, Alipour S (2022). Breast fibroadenomas are less frequent in women with uterine fibroids. Breast Dis.

[8] Shukla P, Misra P, Misra RK (2020). Homoeopathic management of breast fibroadenoma—an open label, single arm, observational trial. Homoeopathic Links.

[9] Khan AT, Shehmar M, Gupta JK (2014). Uterine fibroids: current perspectives. Int J Womens Health.

[10] Commandeur AE, Styer AK, Teixeira JM (2015). Epidemiological and genetic clues for molecular mechanisms involved in uterine leiomyoma development and growth. Hum Reprod Update.

[11] Edzie EK, Dzefi-Tettey K, Brakohiapa EK (2023). Age of first diagnosis and incidence rate of uterine fibroids in Ghana. A retrospective cohort study. PLoS One.

[12] Bewtra C (2009). Fibroadenoma in women in Ghana. Pan Afr Med J.

[13] Ulin M, Ali M, Chaudhry ZT, Al-Hendy A, Yang Q (2020). Uterine fibroids in menopause and perimenopause. Menopause.

[14] Zhao H, Zhou L, Shangguan AJ, Bulun SE (2016). Aromatase expression and regulation in breast and endometrial cancer. J Mol Endocrinol.

[15] Almatrafi MI, Almalki MA, Althagafi JA, AlSindi TS, Masarit RM, Almatrafi RM (2022). Benign breast disease in Makkah, Saudi Arabia: a retrospective analytical cross-sectional study. Cureus.

[16] Klinger K, Bhimani C, Shames J, Sevrukov A (2019). Fibroadenoma: from imaging evaluation to treatment. J Am Osteopath Coll Radiol.

[17] Bhettani MK, Rehman M, Altaf HN, Ahmed SM, Tahir AA, Khan MS, Imran T (2019). Correlation between body mass index and fibroadenoma. Cureus.

[18] Barrett ES, Tran V, Thurston SW, Frydenberg H, Lipson SF, Thune I, Ellison PT (2015). Women who are married or living as married have higher salivary estradiol and progesterone than unmarried women. Am J Hum Biol.

[19] Fortin C, Flyckt R, Falcone T (2018). Alternatives to hysterectomy: the burden of fibroids and the quality of life. Best Pract Res Clin Obstet Gynaecol.

[20] Freytag D, Günther V, Maass N, Alkatout I (2021). Uterine fibroids and infertility. Diagnostics (Basel).

[21] Li J, Humphreys K, Ho PJ (2018). Family history, reproductive, and lifestyle risk factors for fibroadenoma and breast cancer. JNCI Cancer Spectr.

